# Attribution and Expression of Incentive Salience Are Differentially Signaled by Ultrasonic Vocalizations in Rats

**DOI:** 10.1371/journal.pone.0102414

**Published:** 2014-07-21

**Authors:** Juan C. Brenes, Rainer K. W. Schwarting

**Affiliations:** 1 Institute for Psychological Research, University of Costa Rica, Rodrigo Facio Campus, San Pedro, Costa Rica; 2 Neuroscience Research Center, University of Costa Rica, Rodrigo Facio Campus, San Pedro, Costa Rica; 3 Behavioral Neuroscience, Experimental and Biological Psychology, Philipps-University of Marburg, Marburg, Germany; Simon Fraser University, Canada

## Abstract

During Pavlovian incentive learning, the affective properties of rewards are thought to be transferred to their predicting cues. However, how rewards are represented emotionally in animals is widely unknown. This study sought to determine whether 50-kHz ultrasonic vocalizations (USVs) in rats may signal such a state of incentive motivation to natural, nutritional rewards. To this end, rats learned to anticipate food rewards and, across experiments, the current physiological state (deprived vs. sated), the type of learning mechanism recruited (Pavlovian vs. instrumental), the hedonic properties of UCS (low vs. high palatable food), and the availability of food reward (continued vs. discontinued) were manipulated. Overall, we found that reward-cues elicited 50-kHz calls as they were signaling a putative affective state indicative of incentive motivation in the rat. Attribution and expression of incentive salience, however, seemed not to be an unified process, and could be teased apart in two different ways: 1) under high motivational state (i.e., hunger), the attribution of incentive salience to cues occurred without being expressed at the USVs level, if reward expectations were higher than the outcome; 2) in all experiments when food rewards were devalued by satiation, reward cues were still able to elicit USVs and conditioned anticipatory activity although reward seeking and consumption were drastically weakened. Our results suggest that rats are capable of representing rewards emotionally beyond apparent, immediate physiological demands. These findings may have translational potential in uncovering mechanisms underlying aberrant and persistent motivation as observed in drug addiction, gambling, and eating disorders.

## Introduction

Having affective representations in terms of pleasures and desires is a fundamental part of our subjective experience. Rewards and reward-related stimuli can make us feel good, and remind us how good they were in the past, but also how good they would be if experienced again. Being exposed to reward-related cues may also set a state of readiness for seeking and consuming that reward, even though we have not experienced it for a while or when physiological needs for it have been fulfilled [1]–[4]. In humans, such mechanisms play a critical role in drug addiction and relapse, overeating in obesity, and binge disorders [5]–[7]. The incentive valence of such reward-related stimuli (like places, odors, sounds, and time periods) is mainly determined by the affective experience resulting from preceding intake of that reward [8], [9]. Following Pavlovian learning, sensory reward properties and associated cues are transformed into attractive and desired incentives [4], [10]–[13]. This motivational component of reward is normally referred to as incentive salience [5], [10]. In classical and modern incentive motivation theories, either activation of a “central emotive state”, “expectations about rewards”, or “subjective wanting” have been proposed as critical factors in the process of attributing incentive salience to reward cues [9]–[19]. In non-human animals, especially rodents, incentive motivation has been extensively investigated using traditional behavioral parameters, like nose-poking, lever-pressing, and approach behavior to cues and rewards in Pavlovian, instrumental, and Pavlovian-to-instrumental transfer paradigms [5], [9], [20]. The study of the emotional or affective conditioned responses underlying incentive motivation, however, has received less attention, first, because the study of emotions was disregarded in behavioristic tradition (for review see [10]) and second, due to the lack of direct and more precise measures of such states in animals.

Currently, there is an increasing interest in studying rodent ultrasonic vocalizations (USVs) in basic and clinically-oriented research, since USVs seem to provide a unique avenue to study the putative affective state of an animal which might not be accessible by conventional behavioral approaches. Rat USVs are complex affective and communicative signals expressed in different social and non-social situations, which vary according to age and context [21], [22]. Out of these, high-frequency calls (i.e., 50-kHz calls) are normally emitted in naturalistic rewarding situations such as mating, and rough-and-tumble play, or triggered by non-naturalistic stimuli such as electrical stimulation of the mesolimbic dopamine pathways, or by psychostimulant drugs like amphetamine and cocaine [23]–[28]. For instance, individual differences in incentive salience attribution to food cues predicted conditioned place preference for cocaine and 50-kHz calls induced by cocaine related cues [29]. Whether 50-kHz calls may be indicative of incentive salience attributed to food cues, however, is still unclear: previous studies showed mixed results and were not conclusive due to the lack of proper controls groups and concomitant behavioral confirmations of incentive learning [23], [30]–[32].

Encouraged by the translational potential of modeling subjective putative affective states in animals we decided to perform a series of studies to explore further the hypothesis that 50-kHz calls can come to signal a state of incentive motivation in the rat, which may constitute an emotional reward representation triggered by conditioned stimuli (CS) predicting reward or by some perceptual features of the food itself (unconditioned stimulus, UCS). One of the simplest conceivable tests to achieve this aim was training rats to anticipate their daily feeding taking place under certain predictable environmental cues. Within or across experiments the conditioning task was systematically modified so that the current physiological state of the subject (deprived vs. sated), the type of learning mechanism recruited (Pavlovian vs. instrumental), the hedonic properties of UCS (low vs. high palatable food), and the availability of the food reward (continued vs. discontinued) were manipulated.

## General Methods

### Ethics statement

All experimental procedures were approved by the appropriate governmental agency (Regierungspräsidium, Giessen, Germany) and complied with the EU directive 86/609/EEC. Every effort was made to minimize the number of animals used and their suffering.

### Subjects

Adult male Wistar rats (Harlan-Winkelmann, Netherlands) served as subjects. They were housed 4–5 per polycarbonate cage (595×380×200 mm) in a climate-controlled room with a 12:12 h light–dark schedule (light on at 07:00 h). Food (Altromin, Lage, Germany) and water (0.0004% HCL-solution) were freely available unless otherwise specified. In all experiments, animal order of testing was counterbalanced within and across days and experiments to the fullest extent possible.

### Screening cage test

Since rats show substantial and rather stable inter-individual variability in 50-kHz calls [33], we applied a screening test in which rats are tested for their levels of spontaneous USVs before being assigned in a counterbalanced order to further tests or treatments [27], [33]. Briefly, at the beginning of each experiment all animals' cages were removed from the housing-rack and placed on a desk in the same room. Afterwards, a given rat was individually placed into a clean polycarbonate cage (425×266×185 mm) with fresh bedding (Tapvei) and then transported to an adjacent ultrasonic lab, where a recording session immediately started. The cage was placed on a desk under a microphone positioned at 35 cm above the center of cage floor. It was illuminated by red light (about 7 lx inside the cage) and visually separated from the data acquisition area by a curtain. The cage test was conducted on two consecutive days (5 min each). Testing took place from 8:00 to 17:00 h in a counterbalanced order between days and subjects. Based on the average number of spontaneous 50-kHz calls on both days, animals were equally assigned either to the control or the reward group.

### Appetitive cage test

All test settings and the general procedure were the same as described in the screening cage test. Briefly, a given rat was put into a clean cage with bedding, which was then placed on a desk under the microphone, where the recording session immediately started. Two loudspeakers (Avemaster 60 PC stereo system, Germany) connected to a personal computer were placed on either side of the cage. As the conditioned stimulus (CS), a 3-kHz tone (49.2 dB inside the cage) was used. The unconditioned stimulus (UCS) was either normal rat chow (about 20 g) or sweetened condensed milk (10% concentrated milk diluted 1:3 in tap water, Milbona, Germany). For the reward groups, the CS predicted either the start of each daily feeding session (1.5 h access to food per day) or a 30 min-drinking time (milk). Throughout the whole experiment, reward intake took place in the same testing cage used for a given rat. During the first 120 s, animals were left undisturbed (“context” phase), then the CS was presented over another 120 s, subsequently followed by the UCS (food or milk). The overlapping CS-UCS period lasted 30 s once reward intake started. When the tone ended, the animal was allowed to continue consuming the reward for another 60 s before being transported back (in the same testing cage) to the adjacent animal room. A matched control rat was tested simultaneously in a test cage, where it received the same pairing schedule as the matched reward rat did, except that food or milk were never delivered there. Afterwards, the pair of control and reward animals was brought back to the animal room and placed on a rack, with controls on odd and reward rats on even rows, so that both group cages were never side by side. Each control rat remained in its own testing cage while the matched reward rat completed either the 1.5 h-feeding session or 30-min drinking time. At least 3 h after all controls rats had been brought back into their own group cages, namely once the night cycle entered, their 1.5 h-daily feeding session began. In the milk experiments (3 and 4), all animals were first habituated to the sweetened condensed milk for a week. During this period, controls rats had milk in the evening together with their daily food, whereas reward rats had milk in the light period, coinciding exactly with the daily time in which they would be further tested.

### Runway maze

The apparatus was a single U-shaped runway maze constructed of black acrylic, which consisted of two arm alleys (50 cm L×20 cm W×24 cm H) connected by a 20 cm L corridor. The start box (40 cm L) was equipped with a guillotine door that was manually lifted from afar using a pulley cable. The maze was placed on a desk under a microphone held at 45 cm above the center of maze floor. At the distal wall of the goal box, a door was positioned, through which the rat could enter a cage. A second microphone was affixed at 35 cm above the center of the cage floor. The maze was thoroughly cleaned between trials and subjects with a 0.1% acetic acid solution. The testing area was illuminated by red light (about 10 lx inside the maze) and surrounded by curtains.

### Behavioral analysis

Behavior was recorded with a video camera positioned at a longitudinal side of the cage. In Experiment 2, an additional camera recorded behavioral activity in the runway maze. Locomotion (i.e., the number of cage-halves crossed with three paws or the number of 20-cm segments crossed in the runway maze), rearing frequency (i.e., the number of upright postures sustained with hind–paws on the floor), digging (moving cage bedding with forepaws and snout, in seconds), eating or drinking time (seconds), and latency to consume the reward (i.e., time difference between the presentation of food or milk and the first eating or drinking bout, in seconds) were manually counted from videotapes using the EthoLog 2.25 software (University of São Paulo, Institute of Psychology SP, Brazil). Fluid intake (experiments 3 and 4) was determined by weighing bottles before and after testing.

### Ultrasonic recording and analysis

As previously reported [27], USVs were monitored with an UltraSoundGate Condenser Microphone (CM16; Avisoft Bioacoustics, Berlin, Germany) and recorded with Avisoft Recorder 2.7 software (sampling rate: 214,285 Hz; format: 16 bit). High resolution spectrograms (frequency resolution: 0.488 kHz, time resolution: 0.512 ms) were obtained after fast Fourier transformation (512 FFT-length, 100% frame, Hamming window, 75% time window overlap), by using the Avisoft SASLabPro 4.38 software. Experienced observers manually counted USVs off-line from the spectrograms. All USVs emitted over 33 kHz were considered as 50-kHz calls. If two call elements were at least 0.048 s apart, two independent calls were counted. USVs occurring during the tasks were expressed as the number of calls emitted per time (calls/min), except otherwise specified. In the cage tests, the proportion of calls emitted during the tone presentation was expressed as: [(call number during tone/total call number) ×100]. The analysis of 50-kHz calls subtypes (e.g., flat, step-calls, trills) provided no relevant information as groups showed rather similar distributions of such USVs subtypes (data not shown). Therefore they were not included in the analyses and only total call number is presented. Since 22-kHz calls were only rarely and non-systematically observed, they were also omitted from the study.

### Statistical analysis

Results were expressed as means ± SEM. The main effect of groups (G, control vs. reward), training days (D), and their interaction (DxG) was assessed by means of independent mixed ANOVA analyses. In one of the analyses, the repeated-measures factor was the food deprivation days, and in the other, the food ad libitum days. When only one group provided data (i.e., latency to eat and time spent eating in Experiments 1, 2, and 5), a repeated-measures ANOVA within the reward group was computed. All multiple comparisons among days were adjusted with the Bonferroni *post hoc* test. In all repeated-measures analyses that did not meet the sphericity assumption, the Greenhouse-Geisser correction was used. When appropriate, *t*-tests for related samples were used to compare feeding phases within groups. Statistical significance was defined as *p*<.05.

## Results

### Experiment 1

#### Introduction

The hypothesis that 50-kHz calls can come to signal a state of incentive motivation to food reward was investigated by training deprived rats to anticipate their daily feeding. In this experiment the CS signaled the start of each feeding session (1.5 h access to food per day), which began in the ultrasonic lab (∼2 min in the testing cage) and ended in the animal room (see general materials and methods for details). A reward-unpaired rat (i.e., controls) was tested simultaneously in an adjacent room, where it received the same pairing schedule as the matched reward rat, except that a hopper of chow pellets was never placed upon the cage grid.

#### Methods

Thirty experimentally naïve rats weighing 277–351 g on arrival were used. One week before testing, animals were habituated to the experimental conditions and were handled during four days (5 min each). Afterwards, two consecutive screening cage tests were conducted (see screening cage test). Subsequently, animals were counterbalanced into two groups and put on a 22.5-h food deprivation (FD) schedule by being given free access to their maintenance diet for 1.5 h per day, starting one week before the appetitive cage test. During this period, rats were handled and weighed every other day. From day 1 to 7, animals were food deprived (FD); thereafter (days 8–10), they obtained food ad libitum (FAL) in their own home cages.

#### Results

**Figure 1:**
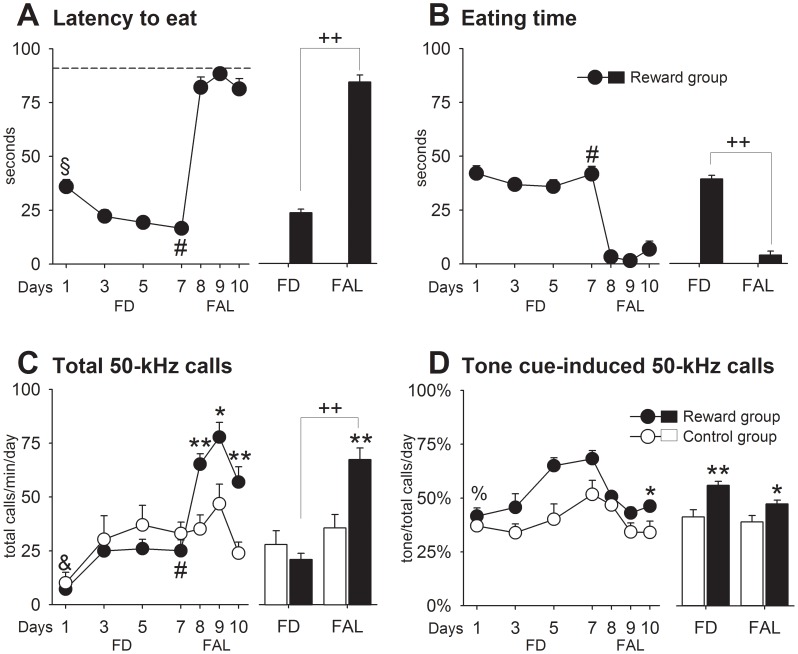
Experiment 1. Animals learned to associate incentive Pavlovian cues with access to daily feeding as reward. A. Latency to eat. B. Eating time. C. Total 50-kHz calls. D. Tone cue-induced 50-kHz calls. The dashed line indicates that a maximal latency time was set at 90 s. Control vs. reward: * *p*<.05, ** *p*<.01. FD vs. FAL: ++ *p*<.01. Day 1 differed from days 5 and 7 in reward rats: § *p*<.05. Day 7 differed from days 8 to 10 in reward rats: # all *p*<.05. Day 1 differed from days 3 to 7 in both groups: & *p*<.05. Data are expressed as mean+SEM (control = 10, reward = 20).

*FD phase*: Reward rats (Fig. 1; days 1–7) showed typical motivational behavior, i.e. approach and food consumption. The latencies to approach the reward decreased over days (D: *F*_3,57_ = 9.57, *p* = .0001). Locomotor activity was lower in reward rats than in controls (G: *F*_1,28_ = 9.24, *p* = .005), whereas rearing activity did not differ between groups (Figure S1). Total call number increased over days (D: *F*_3,84_ = 12.79, *p* = .0001), but contrary to our expectations this effect was observed in both groups (G: *p*>.05) (Figure 1C). The relative number of calls emitted during tone presentation (Figure 1D) did yield a higher percentage of tone-related calls in reward rats (G: *F*_1,28_ = 17.08, *p* = .0001), which increased over days (D: *F*_3,57_ = 9.84, p = .0001), indicating that the reward animals did learn the associations. *FAL phase:* After testing on day 7, animals received food in their home cages in order to devalue the food reward. If the previous lack of group differences seen on total call number was unrelated to incentive learning, no changes in USVs would be expected, but if part of the USVs in reward rats was emitted in anticipation of food, then satiation should decrease them. Surprisingly, we found that total call number increased in reward animals once they were sated (FD vs. FAL: *t*_19_ = −13.10, *p* = .0001), differing now from controls on all FAL days (G: *F*_1,28_ = 13.09, *p* = .001). For instance, call rate in the reward group on day 8 exceeded both, their own levels of day 7 (160%), and controls levels (86%) on the same day (Figure 1C). After 48 h of FAL (i.e. on day 9), total 50-kHz calls reached a maximum, that is, an elevation of ∼310% and ∼166% over FD and control levels, respectively. In contrast, the proportion of calls emitted during tone presentations dropped, especially in the reward group, but was still significantly higher in reward rats (G: *F*_1,28_ = 10.51, *p* = .003). Rearing behavior (Figure S1B), which occurred mostly at the cage side where the food reward was delivered (details not shown), increased in reward rats (FD vs. FAL: *t*_19_ = −4.89, *p* = .0001) and remained consistently high until the end of the FAL phase (G: *F*_1,28_ = 11.04, *p* = .002). Remarkably, the increases in appetitive 50-kHz calls and rearing occurred even though approach and consummatory behaviors were completely abolished during all FAL days (Figure 1A and B). Thus, the devalued feeding conditions dramatically increased both total call number and tone-induced USVs even after 72 h of experiencing the reward in a low motivational state.

#### Discussion

These data suggest that attribution of incentive salience to reward predictive cues (i.e., cage context and tone CS) may have occurred while animals were deprived, and surprisingly cues were able to trigger conditioned motivational reactions to reward, i.e. USVs, even though it was fully devalued. Since the conditioned response was learned under FD, expression but not acquisition of such a response is what seemed to be suppressed during this phase. Perhaps, approach/consummatory responses taking place in the same testing environment may have overlapped with the preparatory/emotional elements of the UCS producing a sort of inhibition in the expression of the latter [34]. For example, species-specific foraging behavior such as digging/snout-down locomotion, which was also observed here (Table S1, supporting information) is known to increase during FD even when animals never obtained food by these means [35], [36]. Since these and other approach/consummatory behaviors were reduced drastically when tested under FAL conditions, we consider the idea of competition between different behavioral systems.

### Experiment 2

#### Introduction

Here, the procedure was modified so that USVs associated with anticipatory and consummatory acts could be measured in different testing compartments. A testing cage with bedding was also used here, but instead of training animals to passively wait for food reward to be delivered (i.e., Pavlovian schedule), they now learned to run down a runway maze connected to the cage, so that they could voluntarily enter it and access their daily food ration available there (i.e., instrumental component). With these modifications, animaĺs motivation to attain the reward and cue-induced anticipatory 50-kHz calls in the runway could be assessed independently from consummatory responses in the goal cage. As anticipating and earning a reward appear to be distinct processes [8] we sought to elucidate whether the USV effects observed in experiment 1 rely upon the type of associative process. The cage was the same as in Experiment 1, so that each animal had its own cage for testing throughout the whole experiment.

#### Methods

The same 30 rats used in Experiment 1 served as subjects, weighing 361–440 g at the beginning of this experiment, which took place 27 days apart from the first experiment. Exactly as there, all animals were put on a 22.5-h schedule of FD with free access to their maintenance diet (1.5 h per day) either immediately after testing (for reward animals in their own testing cages) or at least 3 h later (for controls once they were returned to their group cages). After 3 handling days, habituation to the runway was begun. This consisted of taking the rats from their home cages and placing them in pairs into the start box of the maze (with the door opened) for about 15 min during three consecutive days. In parallel, we performed reinstatement of tone/food pairing by repeating the cage test procedure of Experiment 1. During seven days, starting from the second day of the runway habituation, animals were given a maze habituation session followed by a cage test procedure. On the next two days, both procedures were combined, that is, single animals were placed into the maze with the cage attached to it (with food for reward rats). When they entered the cage, a 3-kHz tone was played as in Experiment 1. During habituation, animals were weighed and handled every other day. Afterwards, reward animals were trained to run through the runway maze to access food in the cage attached to the end of the runway goal arm. Rats received daily training sessions for 10 consecutive days conducted as follows: A given rat was confined to the start box for 120 s, and during the last 60 s a 3-kHz tone was played, which ended with opening of the door. Afterwards, rats were free to locomote between runway and cage during approximately 4 min. Control rats followed the same procedure but food was never given in the cage. As in Experiment 1, animals were food deprived during days 1–7; thereafter (days 8–10) they received FAL in their own home cages. USVs were recorded during the entire testing period, since animals used to shuttle between runway and cage.

#### Results

**Figure 2:**
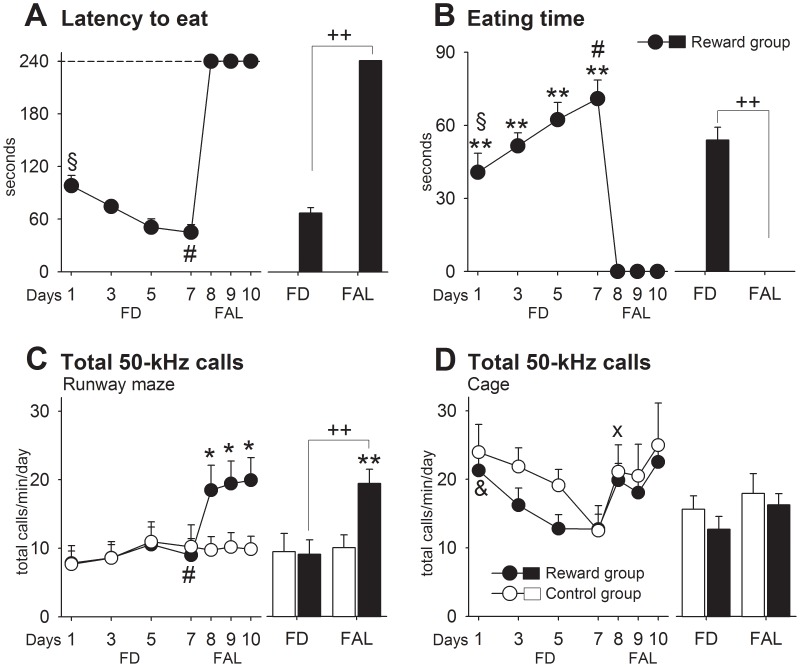
Experiment 2. Animals learned to access their daily feeding in a cage by running through a runway maze attached to it. A. Latency to eat. B. Eating time. C. Total 50-kHz calls in the runway maze. D. Total 50-kHz calls in the cage. The dashed line indicates that a maximal latency time was set at 240 s. Control vs. reward: * *p*<.05, ** *p*<.01. FD vs. FAL: ++ *p*<.01. Day 1 differed from days 5 and 7 in reward rats: § *p*<.05. % Day 1 differed from days 3 to 7 in reward rats: § *p*<.05. Days 1 and 3 differed from day 7 in both groups: & *p*<.05. Day 7 differed from days 8 to 10 in reward rats: # all *p*<.05. Day 7 differed from days 8 and 10 in both groups: x all *p*<.05. Data are expressed as mean+SEM (control = 10, reward = 20).

*FD phase:* As shown in Figure 2, the latencies to eat declined (D: *F*_3,57_ = 8.43, *p* = .0001) and eating times increased over days in the reward group (D: *F*_3,57_ = 5.03, *p* = .004). In the runway maze, locomotion (D: F_3,84_ = 26.79, *p* = .0001) and rearing (D: F_3,84_ = 90.17, *p* = .0001) but not USV (D: *p*>.05) decreased over days (Figure S2A, S2B, and 2C, respectively). For all these variables, no group differences were observed (G: *p*>.05). In the cage, all animals emitted more 50-kHz calls per time than in the maze (Figure 2D). There, USVs diminished over days (D: *F*_3,84_ = 9.67, *p* = .0001) without differing between groups (G: *p*>.05). Cage locomotion (G: *F*_1,28_ = 44.93, *p* = .0001) and rearing (G: *F*_1,28_ = 18.89, *p* = .0001) were higher in controls (Figure S2C and S2D), perhaps since reward rats were now engaged in eating while controls still explored the cage. *FAL phase:* As in experiment 1, rats received FAL after testing on day 7. Again, the latencies to eat increased (FD vs. FAL: *t*_19_ = −29.64, *p* = .0001) and eating times decreased in the reward group (FD vs. FAL: *t*_19_ = 10.31, *p* = .0001). In contrast, and consistent with experiment 1, reward cues associated to the runway maze now elicited enhanced 50-kHz calls (Figure 2C). For instance, total call number in reward rats was now ∼210% higher compared with their own USVs levels while FD (FD vs. FAL: *t*_19_ = −13.10, *p* = .0001), and ∼195% higher than that in controls on all FAL days (G: *F*_1,28_ = 7.07, *p* = .01). In the cage (Figure 2D), 50-kHz calls increased in both groups (D: *F*_3,84_ = 4.72, *p* = .004), but did not differ from each other (G: *p*>.05). There were no group differences in locomotion or rearing (G: *p*>.05) (Figure S2).

#### Discussion

This experiment replicated the results from Experiment 1 in which no differences in total call number were observed under FD, but increased USVs occurred in reward animals once they became sated. The suppressive effect of FD probably did not depend on the type of learning recruited, the behavioral competition between approach/consummatory and anticipatory affective responses, or the interference of an opposite behavior such as digging since the maze had no bedding. Since animals were free to shuttle between maze and cage and most reward animals revisited the maze between eating bouts (details not shown), maze cues were not just temporally predicting further access to reward, but also became imbued with incentive salience after animals re-experienced the UCS, facilitating CS representations to be re-updated within and across training days. As a consequence, runway maze cues, but not cage cues, triggered appetitive 50-kHz calls suggesting that USVs were specifically sensitive to the type of learning mechanism recruited. Again, attribution of incentive salience to food cues seemed to take place during the FD period, whereas the expression of such an appetitive response occurred once animals became sated, that is, when the reward was devalued and when no other appetitive behaviors were emitted. Finally, the analysis of USVs in the cage revealed that satiation on its own increased 50-kHz calls irrespective of being food rewarded or not.

### Experiment 3

#### Introduction

As just shown, the current physiological state of the rat produces a biphasic expression of 50-kHz calls in reward rats, which according to Experiment 2 is not dependent on the interference between preparatory and consummatory responses [16] or the competition between species-specific responses activated by the UCS (e.g., foraging inhibiting calling, for review see [37]). To simplify data collection and analysis we went back to the test settings of Experiment 1. Now, we asked whether increasing the incentive properties of the reward would enhance motivation for UCS during the FD period. To this end, a high palatable reward (i.e., sweetened condensed milk) was used. Since the reward delivered in the testing cage was different from normal rat chow, access to reward became independent from the daily feeding session. Thus, we expected that it could still be valuable when testing animals under satiation.

#### Methods

Twenty-four experimentally naïve rats weighing 231–256 g on arrival were used. Habituation to the animal facilities, handling, the screening cage test, and FD schedule were conducted as in Experiment 1, but now, the CS signaled access to a 30 min-drinking period: ∼2 min in the cage and the remaining time in the animal room. The reward group had access to sweet condensed milk, whereas the control group had access to tap water. One week before testing, rats were habituated to sweetened condensed milk. During this period, all rats were handled and weighed every other day. Testing was performed with the former FD/FAL schedule: FD from day 1 to 7, and FAL thereafter (days 8–10).

#### Results

**Figure 3:**
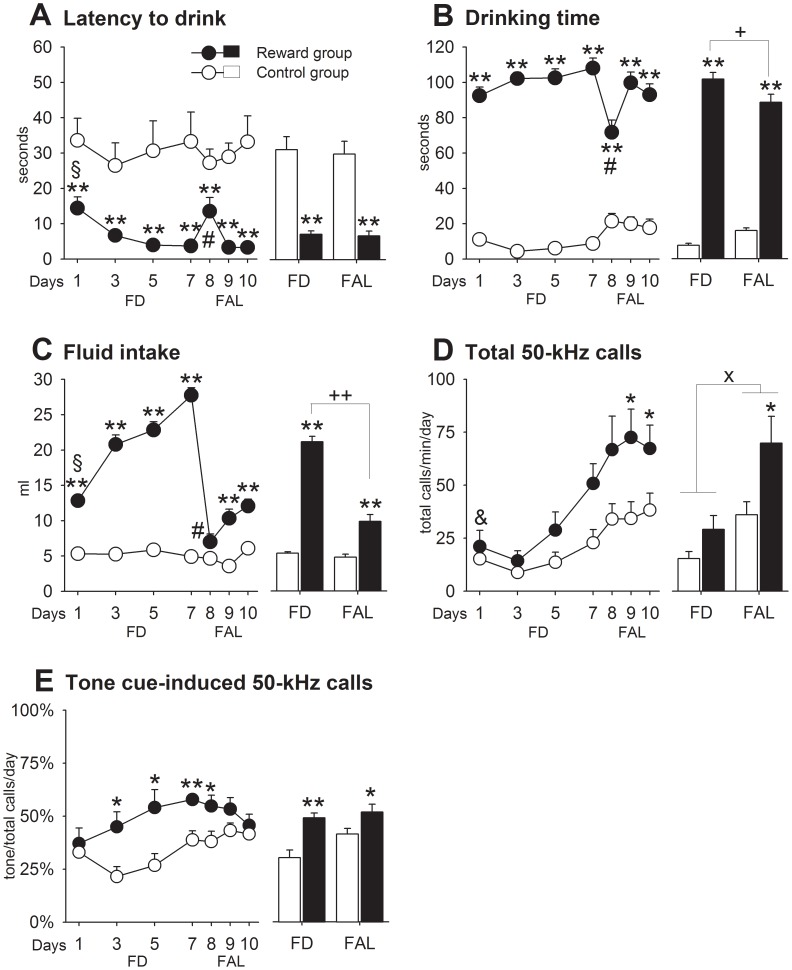
Experiment 3. Animals learned to associate incentive Pavlovian cues with access to sweetened condensed milk as reward. A. Latency to drink. B. Drinking time. C. Fluid intake. D. Total 50-kHz calls. E. Tone cue-induced 50-kHz calls. Control vs. reward: * *p*<.05, ** *p*<.01. FD vs. FAL: + *p*<.05, ++ *p*<.01. FD vs. FAL in both groups: x all *p*<.05. Day 1 differed from days 3 to 7 in reward rats: § *p*<.05. Day 1 to 5 differed from day 7 in both groups: & *p*<.05. Day 8 differed from days 7, 9, and 10 in reward rats: # all *p*<.05. Data are expressed as mean+SEM (control = 12, reward = 12).

*FD phase:* The latencies to drink (Figure 3) diminished slightly once training began, with reward rats being ∼6 times faster than controls, which were given a bottle with tap water (G: *F*_1,22_ = 85.05, *p* = .0001; DxG: *F*_3,66_ = 16.34, *p* = .002). The times spent drinking (G: *F*_1,22_ = 538.80, *p* = .0001; DxG: *F*_3,66_ = 15.78, *p* = .0001) and daily milk consumption (G: *F*_1,22_ = 582.71, *p* = .0001; DxG: *F*_3,66_ = 4.19, *p* = .009) were higher in the reward group where they increased also over days. Total call number augmented over days in all animals (D: *F*_3,66_ = 12.05, *p* = .0001), especially after the third day (Figure 3D), but did not differ significantly between groups (G: *p*>.05). In contrast, the percentage of tone-induced 50-kHz calls was higher in the reward group (G: *F*_1,22_ = 19.05, *p* = .0001) (Figure 3E). *FAL phase:* When tested without deprivation, there was a transitory increase in the latency to drink and a transitory reduction in the time spent drinking which fully recovered on the following FAL days. Despite such small variations when feeding conditions changed, reward rats still differed from controls on latencies to drink (G: *F*_1,22_ = 76.23, *p* = .0001) and times spent drinking (*F*_1,22_ = 76.23, *p* = .0001). Milk intake declined drastically (FD vs. FAL: *t*_11_ = −11.41, *p* = .0001), almost reaching control levels on the first FAL day, but was higher than controls again thereafter. On the following FAL days, milk intake partially recovered between about 25% to 43% of the maximal intake achieved under FD (G: *F*_1,22_ = 17.08, *p* = .0001; DxG: *F*_3,66_ = 10.03, *p* = .0001). In addition, locomotion (FD vs. FAL: *t*_11_ = −8.45, *p* = .0001) and rearing (FD vs. FAL: *t*_11_ = −9.01, *p* = .0001) increased in the reward group and exceeded those of controls (locomotion, G, *F*_1,22_ = 758, *p* = .01; rearing, G: *F*_1,22_ = 18.06, *p* = .0001; Figure S3A and S3B). Similar to Experiments 2 and 3, call rate was potentiated by shifting the feeding conditions, an effect which now occurred in both groups (D: *F*_3,66_ = 3.14, *p* = .03). Interestingly, the attenuation of approach and consummatory behaviors observed when shifting feeding conditions was not paralleled by a reduction in total call number and percentage of cue-induced calls. Instead, total call number (Figure 3D) was now significantly higher in the reward group (G: *F*_1,22_ = 6.60, *p* = .02), and tone cue-induced calls were also higher (G: *F*_1,22_ = 5.18, *p* = .03) but returned towards control levels over days (Figure 3E).

#### Discussion

Relative to previous experiments, USVs appeared slightly increased at the end of the FD period, although no overall effect on total call number was detected. However, the percentage of tone-induced calling increased during FD indicating that conditioning strengthened over days. Once again, reward devaluation decreased neither total call number nor tone-induced calling, as it had initially been expected. Instead, total call number increased while cue-induced USVs remained slightly high during FAL days. Regarding reward palatability, the higher incentive properties of the UCS (i.e., milk) plus the likely invigorating effect of the CS seemed to maintain latencies to drink and times spent drinking in the cage while the UCS was degraded in agreement with previous reports [1], [3], [4]. However, the amount of milk intake, most of which was consumed in the animal room without the influence of the CS, appeared to be drastically reduced by satiety.

### Experiment 4

#### Introduction

So far, the increased percentage of 50-kHz calls induced by the tone cue indicated that attribution of incentive salience to reward-related stimuli had successfully taken place during FD, even thought it was not clearly translated into an overall elevation of 50-kHz calls. To account for such an inhibition in USVs utterance, we assume that the ability of food CS cues to elicit appetitive 50-kHz calls was possibly suppressed by FD, an effect that occurred independently from learning acquisition. So far, the three preceding experiments showed that restoring FAL feeding conditions after FD increased spontaneous USVs in controls and potentiated total call number and food cues-induced appetitive 50-kHz calls in reward rats. This may suggest that FD itself was able to suppress USVs particularly at the time when animals were expecting the food reward. Having access to food after a long FD period recruits not only positive but also negative reinforcement mechanisms, and may be described as a transition from distress to pleasure [38]. FD can induce an aversive state so that animals will work to prevent starvation by either prolonging the period of food availability [39] or escaping from a CS signaling the omission of an expected food reward [40]. This evidence raised the question of whether the same palatable reward (i.e., milk), now acquired in the absence of FD, would be sufficient to increase appetitive 50-kHz calls.

#### Methods

Twenty experimentally naïve rats weighing 259–279 g on arrival were used. The experimental procedure was generally the same as in Experiment 3, with sweetened condensed milk also used as reward. However, contrary to all previous experiments, the acquisition phase (days 1–7) of UCS-CS pairing occurred first in the FAL phase and was followed by the FD phase (days 8–10).

#### Results

**Figure 4:**
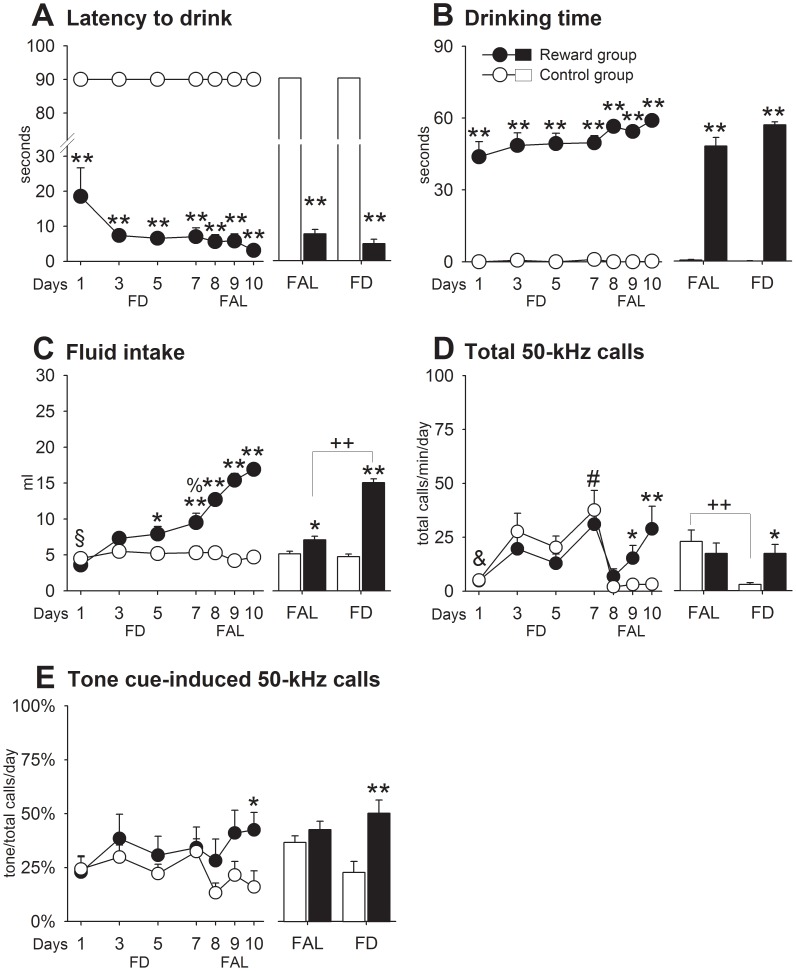
Experiment 4. Animals learned to associate incentive Pavlovian cues with access to sweetened condensed milk as reward. A. Latency to drink. B. Drinking time. C. Fluid intake. D. Total 50-kHz calls. E. Tone cue-induced 50-kHz calls. Control vs. reward: * *p*<.05, ** *p*<.01. FAL vs. FD: ++ *p*<.01. Day 1 differed from days 5 and 7 in reward rats: § *p*<.05. Day 1 differed from days 3 to 7, and day 5 differed from day 7 in both groups: & *p*<.05. Day 7 differed from days 8 to 10: % *p*<.05. Day 7 differed from all FD days in controls, and from day 8 in reward rats: # all *p*<.05. Data are expressed as mean+SEM (control = 10, reward = 10).

*FAL phase:* As expected, reward rats showed shorter latencies to drink (G: *F*_1,18_ = 1252.46, *p* = .0001) and more time spent drinking (G: *F*_1,18_ = 172.56, *p* = .0001) than controls which consumed almost none of the tap water (Figure 4A and B). As soon as reward animals had learned that milk was available, latencies and drinking times did not change over the FAL days (D: *p*>.05). However, the amount of milk (Figure 4C) consumed augmented with repeated testing (G: *F*_1,18_ = 8.02, *p* = .01; DxG: *F*_3,54_ = 4.05, *p* = .01). Total call number increased over days (D: *F*_3,54_ = 24.86, *p* = .0001), with no significant differences between groups (G: *p*>.05) (Figure 4D). Likewise, no differences were observed for the percentage of tone-induced calling (Figure 4E). Also, exploratory activity appeared unaffected by both, repeated testing and reward experience (Figure S4A and S4B). *FD phase:* Latencies to drink (G: *F*_1,18_ = 147.51, *p* = .0001) and times spent drinking (G: *F*_1,18_ = 2039.35, *p* = .0001) remained higher in the reward group compared to controls (Figure 4A–B). The more noticeable effect of FD occurred on the amount of milk consumed, which scaled up between 34% to 78% over preceding FAL levels (FD vs. FAL: *t*_9_ = −11.47, *p* = .0001; Figure 4C), whereas water intake remained unaffected (G: *F*_1,18_ = 190.58, *p* = .0001). Contrary to approach and consummatory behaviors, call rate dropped drastically on the first FD day in both groups (Figure 4D) (D: *F*_3,54_ = 16.58, *p* = .0001). On the following FD days, total call number progressively returned to FAL levels only in the reward rats (DxG: *F*_3,54_ = 3.74, *p* = .03). Similarly, the percentage of tone-induced USVs was significantly higher in the reward group (G: *F*_1,18_ = 11.82, *p* = .003) (Figure 4E). Locomotion (FD vs. FAL: *t*_9_ = 3.31, *p* = .009) and rearing behavior (FD vs. FAL: *t*_9_ = 5.96, *p* = .0001) were reduced in controls (Figure S4), whereas in reward rats only locomotor activity was reduced by FD (FD vs. FAL: *t*_9_ = 2.96, *p* = .02).

#### Discussion

This experiment shows first, that a highly palatable food reward was not sufficient to increase either total call number or tone cue-induced calling on its own and, second, that FD was able to suppress calling in both groups. Since the reward did not have a high hedonic value during the acquisition phase (i.e., when tested under FAL conditions), total call number and cue-induced 50-kHz calls recovered and differed from control levels only when the hedonic representation of that reward was updated while in the subsequent state of being hungry. This is consistent with studies showing that the instrumental response guided by previous reward expectations changes only when the new hedonic value of the incentive is experienced [8], [9]. In this as well as in previous experiments, the motivational state of being FD seems to be required for reward cues to be imbued with incentive salience, even though it suppressed overall USVs utterance.

### Experiment 5

#### Introduction

The likely aversive state provoked by long FD may have accounted for some suppressive effects in calling in our previous experiments. In experiment 3, however, total call number tended to increase while FD, an effect that, according to Experiment 4, may not solely be attributed to palatability. Another factor might be critical: By replacing rat chow with milk as a reward we also inevitably altered the predictive association between access to reward in the cage and daily feeding session. If expectations about reward were controlled by the very first access to food or milk (2 min) –and not by the whole period of eating (1.5 h) or drinking (30 min)–, it is very likely that animals learned to anticipate the short access to reward instead of the long one. Thus, when animals encountered the reward, a negative discrepancy between the reward expected and the one actually obtained may have been experienced, an effect probably energized by FD. We hypothesized, therefore, that providing continued access to reward in the testing environment would prevent such negative discrepancy to occur, ‘releasing’ the expression of reward-related appetitive USVs when FD. To test this idea, we adapted the procedure of Experiment 1 in which the higher suppression in calling was observed.

#### Methods

Twenty experimentally naïve rats weighing 240–265 g on arrival served as subjects. Habituation to the animal facilities, handling, and the screening cage test were conducted largely as in Experiments 1 and 2: Food pellets (normal rat chow) served as reward, but contrary to there, both access to reward and the completion of the daily feeding session took place exclusively in the testing room. Indeed, during habituation to FD, reward rats had access to the daily food ration only in the testing room, so that the fact of being fed after a 22.5-h FD period was specially linked to this environment. During testing, rats were FD from days 1 to 7. Controls never accessed their daily food ration either in the cage or in the experimental room where testing took place.

#### Results

**Figure 5:**
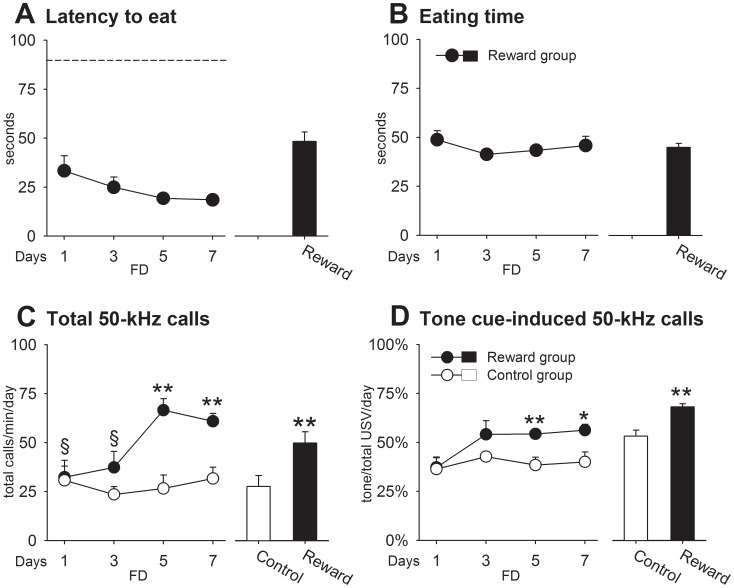
Experiment 5. Animals learned to associate incentive Pavlovian cues with access to daily feeding as reward. A. Latency to eat. B. Eating time. C. Total 50-kHz calls. D. Tone cue-induced 50-kHz calls. The dashed line indicated that a maximal latency time was set at 90 s. Control vs. reward: * *p*<.05, ** *p*<.01. Days 1 and 3 differed from days 5 and 7 in reward rats: § *p*<.05. Data are expressed as mean+SEM (control = 10, reward = 10).

Animals approached the food and started eating without any noticeable change from the beginning to the end of testing (D: *p*>.05) (Figure 5A and B). Contrary to our previous food experiments, reward cues did now increase total call number over FD days (DxG: *F*_3,54_ = 8.19, *p* = .0001) (Figure 5C), and calls were approximately 120% higher than that in controls (G: *F*_1,18_ = 7.54, *p* = .01), which showed rather stable call rates over days. Likewise, the percentage of tone-induced calling was significantly higher in reward rats than in controls (G: *F*_1,18_ = 17.90, *p* = .001) (Figure 5D). Unlike locomotion (Figure S5A), rearing behavior in reward rats showed a progressive increase mirroring the one observed for USVs (G: *F*_1,18_ = 11.07, *p* = .004; DxG: *F*_3,54_ = 9.81, *p* = .0001), yet to a lesser extent (Figure S5B).

#### Discussion

This experiment shows that providing continuous access to reward in the testing environment enables attribution and expression of incentive salience during the FD period, and contrary to previous experiments, this effect was now noted both in terms of total call number and the percentage of cue-induced 50-kHz calls.

## General Discussion

Following Pavlovian incentive learning, a CS for reward comes to produce expectations and potentiates motivation about the UCS. Here, we sought to determine whether rat 50-kHz USVs may signal such a state of incentive motivation to a natural, nutritional reward. In general, we found that reward-cues become effective to elicit 50-kHz calls. Under certain conditions, however, utterance of 50-kHz calls can be either suppressed during a highly motivational state, or more strikingly, can be elicited when food rewards were devalued by satiation. In both cases, changes in calling occurred independently from motivation to approach and consume the reward. Interestingly, this phasic character of USVs was consistently seen across independent experiments.

We acknowledge that the question of whether 50-kHz calls can be elicited by food rewards has already been addressed. The pioneer study [23] showed an increase in anticipatory 50-kHz calls in FD rats (i.e., before daily feeding) at the end of a 6-days period of training. In a sucrose self-administration paradigm conducted with non-FD rats, 50-kHz calls increased over time when comparing different time points throughout a testing period of 4–5 weeks [30]. Others described differences in 50-kHz calls between adolescent and adult rats when offered chocolate chips in a one-trial test [32], and one study yielded differences in calling following positive and negative reward contrasts with different probabilities to obtain sucrose reinforcement [31]. In all these studies, however, it was unclear whether call rate in food-paired rats was different from spontaneous calling mainly because no control rats were included [23], [30]–[32]. It has repeatedly been observed that rats call at moderate rates simply by the fact of placing them in certain testing environments [27], [33], [41]. For example, in the only two studies showing positive results [23], [30] calling increased over days in a similar way as it did in our control rats in Experiments 1 and 4, in which no groups differences were found until feeding conditions were changed. Also, previous studies did not test whether increases in 50-kHz calls were accompanied by changes in other relevant learning or motivational parameters because no concomitant behavioral measures were described [23], [30], [32]. In general, we went beyond those studies showing that USVs in reward rats as compared to matched controls were differentially sensitive to the current physiological state of the subject (FD vs. FAL), the type of learning mechanism recruited (more Pavlovian vs. more instrumental), the hedonic properties of the UCS (low vs. high palatable food), its availability (continued vs. discontinued), and the relation between 50-kHz calls and other behavioral dimensions indicative of incentive learning and conditioning.

**Figure 6:**
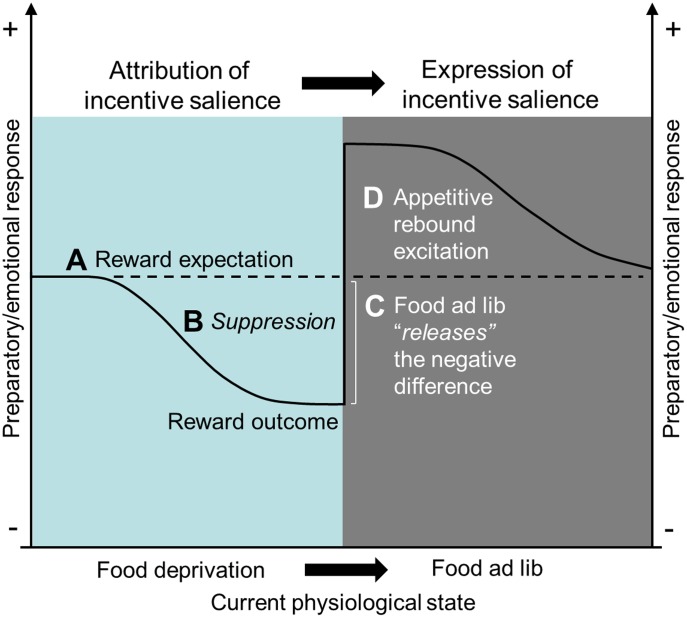
Suppression-release hypothesis. This hypothesis integrates statements modified from previous models [16], [34]. A) If the strength (positive magnitude) of the actual UCS is less than the strength of the subject's expectation (maximized by food FD), the result will be suppression in the expression of the conditioned response. B) The larger the discrepancy between the strength of the expectation and the strength of the UCS outcome, the greater the suppression. C) If the strength of the actual UCS equals the strength of the subject's expectation, no suppression will be observed. Then, the magnitude of the suppression released would be proportionally inversed to the strength of the suppression that precedes it. Restoring the normal feeding conditions acts as a “releasing” factor of the negative difference between reward expectation and outcome, which does not require updating and which occurs afresh as soon as the current physiological state shifts. In the present experiments, the suppression in the expression of the conditioned response cannot be explained in terms of conditioned inhibition, because the UCS was always presented and therefore no CS- signaled its absence. D) The release of a motivational system (i.e., appetitive) from inhibition by the opponent system (i.e., aversive state induced by negative reward expectations) produces a rebound appetitive excitation that may last longer and decay slower for preparatory/emotional conditioned responses than for consummatory/sensorial ones.

**Figure 7:**
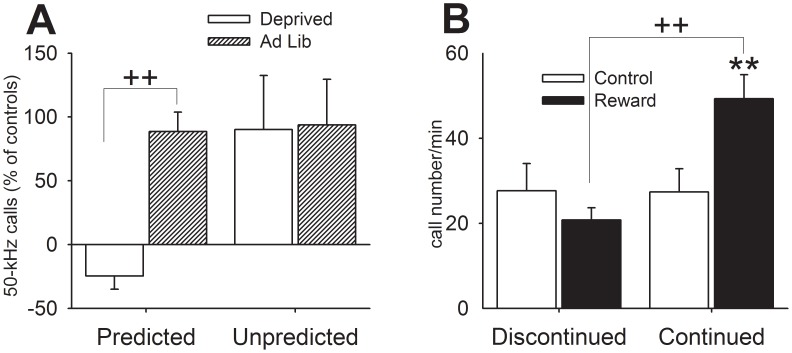
Comparisons between Experiments 1 and 3 (A) and 1 and 5 (B). Access to daily feeding (1.5-h) was predicted by reward in the cage in Experiment 1 (A, left) but not in Experiment 3 (A, right), in which animals received a reward independent of feeding. Access to reward was discontinued (B, left) in Experiment 1 (∼2 min in the cage and the remaining time of 1.5-h period in the animal room). Providing continued access to reward in the testing cage (B, right) prevented the suppression in calling observed in Experiment 3. Control vs. reward: ** *p*<.01. FD vs. FAL interaction: ++ *p*<.01. Data are expressed as mean+SEM.

Our results also make us believe that FD on its own induced a putative negative state that affected USVs likelihood. This assumption is also supported by our previous findings in which FD consistently suppressed spontaneous calling over four consecutive days [33]. Since FD is aversive enough to strongly motivate escape and avoidance responses [38]–[40], it is not surprising that FD exerted a suppressive effect on 50-kHz calls utterance similar to those produced by other aversive stimuli [21], [22], [25]. Still the question remains, how FD particularly suppressed the expression of the emotional conditioned response during acquisition while being FD. In all experiments in which reward intake was discontinued (Experiments 1–4), animals seemed to learn about the association between testing and the 2-min access to reward permitted while they remained in the testing room. Figure 6 depicts a model that summarizes our assumptions in terms of a suppression-release hypothesis, in which we propose that FD played a critical role in the suppression of cue-induced calling by increasing the negative contrast between reward expectation and outcome. As training progressed, the 2-min access to food became gradually insufficient for experiencing the positive effects of consuming the food and therefore the expectation of being fed and becoming sated did not match each other. For example, in the rat chow experiment access to the reward in the cage predicted the 1.5-h feeding session, which was the only feeding opportunity animals had. In the milk experiment, conversely, access to reward in the cage predicted only a 30-min drinking time, which was independent of feeding. Although the duration of daily feeding was adjusted to keep body weight at 90% of baseline based on the caloric income of milk, animals still had two unrelated feeding opportunities as compared to only one in rat chow experiments. Thus, reward expectation should have been proportional to the magnitude and density of reward, being therefore greater when giving chow pellets than when giving milk. To better illustrate this point, calling was compared between Experiments 1 and 3: Figure 7 shows call rate as relative to control levels to normalize the fluctuations in calling due to the FD itself. As can be seen in FD rats receiving normal rat chow, the score for cue-induced calling is negative, namely, 25% lower than spontaneous calling in controls. Once sated, calling increased by 90% over control levels. In rats receiving milk reward, in contrast, the relative increase in cue-induced calling was about 90% over control levels while FD but also in the FAL phase. This indicates that suppression in cue-induced calling depended upon the predictive strength of the reward in relation to feeding: the larger the discrepancy between expectation and UCS outcome, the greater the suppression. Once sated, the magnitude of suppression released was inversed to the strength of suppression that preceded it. We acknowledge that both rewards differed in their incentive properties, but as shown in Experiment 4, palatability on its own is unlikely to account for all differences observed between experiments using chow pellets vs. milk. In addition, we plotted calling results of Experiments 1 and 5 to highlight the contribution of reward expectations on USVs suppression (Figure 7B). When providing continued access to reward while all other factors remained equal, reward expectation matched outcome and thus, suppression was no longer observed. Then, attribution and expression of cue-induced calling occurred simultaneously over FD days. Based on this assumption one might think, even counter-intuitively, that what FAL did was removing the negative expectation of the outcome given by the difference between being hungry and having access to an insufficient reward. Without the urge for food, the outcome matched the expectation of being rewarded. In other words, restoring the normal feeding conditions acted as a ‘releasing’ factor of the negative difference between reward expectation and outcome, which did not require updating and occurred afresh as soon as the current physiological state shifted (for an example of instant transformation of an aversive cue into a desired one see [42]). The suppression in the conditioned response cannot be explained in terms of conditioned inhibition, because the UCS was always presented and therefore no CS signaled its absence. A negative difference between reward expectations and the actual outcome has previously been described in terms of a frustration effect [40], [43] (for review see [44]). Such a reward discrepancy or devaluation can induce a putative, negative affective state able to elicit escape responses [39], [40], intra- and hetero-specific aggression [45], high corticosterone levels [46], and distress USVs. For example, 11-day-old rat pups that had learned to approach an anesthetized dam with dry suckling as a reward showed distress USVs when reward was denied [47]. In adult rats, reductions in 50-kHz calls or increases in distress USVs (i.e., 22-kHz calls) have also been reported following timeout, withdrawal, or devaluation of different rewards [26], [31], [48]. Here, it should be noted that distress calls were only rarely observed in our present experiments.

It is still surprising however, that the updating of hedonic value of the UCS under a state of satiation did not reduce the ability of the CS to induce appetitive 50-kHz calls across experiments. If animals had not experienced the reward during the FAL phase we can perhaps assume that rats were behaving based on previous expectations built up when the reward was still valued [8], [9]. However, some animals in the rat chow experiments approached the food, sniffed it or gnawed it; something that may have been enough to re-update the new hedonic value of that reward, even though none actually ate a piece of food in the cage (it was freely available in home cages). In contrast, in the milk experiment (3) all animals re-experienced the reward during satiation. At least from day 8 to day 9, and from there to day 10, a re-update of the current hedonic value of the food reward should have happened. Nevertheless, it did not impede incentive motivation reactions to occur (i.e., 50-kHz calls and, to a lesser extent, rearing behavior). We acknowledge that the FAL period measured may not have been long enough for a back-to-baseline drop in USVs rate, however, it was apparently sufficient for consummatory behavior to be completely abolished (rat chow experiments) or drastically reduced (milk experiment). We have provided a putative explanation for this effect consistent with the suppression-release hypothesis developed here. According to the Konorskian opponent-process model [16] (e.g., see [9]), we propose that the release of a motivational system (i.e., appetitive) from inhibition by the opponent system (i.e., aversive state induced by negative reward expectations maximized by FD) produced a rebound appetitive excitation, which may have lasted longer and decayed slower for preparatory/emotional responses than for consummatory/sensorial ones [16], [34]. The latter responses are normal unconditioned feeding behaviors that are expected to be rapidly suppressed or activated according to physiological demands. Cue-induced USVs and exploratory activity, in contrast, are acquired conditioned responses controlled more by environmental stimuli rather than by the current appetitive state. It seems quite reasonable that cues that had signaled food availability while in a state of need were still able to guide behavior despite physiological needs were transiently fulfilled, since requirements of food may change as a matter of hours and, therefore, places where it was consistently available should be well remembered. This differential expression of incentive motivation responses may explain why right after satiation 50-kHz calls appeared detached from food seeking and consumption. To our knowledge, there is no such evidence as the one presented here, and as recently reviewed elsewhere [5], persistent incentive motivation has only been described when the UCS was devalued in the absence of the previous CS or following over-training, which was not our issue. Further experiments employing different conditioning paradigms with longer and more diverse testing schedules are required to elucidate the phenomena shown here, especially regarding USVs. However, it seems clear so far that independent of Pavlovian or instrumental task demands, reward palatability, reward accessibility and availability, devaluation of reward did not reduce cue-induced appetitive 50-kHz and rearing behavior, and more strikingly, shifting the current physiological state of the animals, and probably their subjective emotional state too, elicited appetitive 50-kHz calls by the pure incentive salience attributed to the environmental cues of the testing situation.

## Conclusions

After Pavlovian incentive learning, reward-cues became able to elicit 50-kHz calls presumably signaling a state indicative of appetitive incentive motivation in the rat. Attribution and expression of incentive salience, however, do not seem to be a unified process, and were teased apart in two different ways: 1) under a high motivational state (i.e., hunger) the attribution of incentive salience to cues occurred without being expressed at the USVs level; and 2) under a low motivational state (i.e., food satiation), expression of appetitive USVs persisted despite physiological needs being fulfilled. In both cases, putative affective incentive responses were elicited independently from motivation to approach and consume the reward. While in a hungry state, short access to rewards may have led to a discrepancy between the reward expected and the one actually obtained that likely suppressed expression of USVs. When such a discrepancy between reward expectations and outcome was prevented by providing continued access to food, attribution and expression of incentive salience synchronized. Similarly, shifting feeding conditions from deprivation to satiation acted as a ‘releasing’ factor of the putative aversive state induced by both reward discrepancy and food deprivation. Such a release of a motivational system from inhibition led to a rebound appetitive excitation that lasted longer and decayed slower for preparatory/emotional responses than for consummatory/sensorial ones. The latter may explain why appetitive 50-kHz calls increased while sated but detached from reward seeking and consumption. Finally, the fact that rats seem to represent rewards emotionally (for review see, [21]) and beyond apparent, immediate physiological demands, provides an unparalleled translational tool to model motivational mechanisms underlying eating disorders, and may even be extendable to other forms of aberrant or persistent motivation such as in drug addiction, or gambling disorders.

## Supporting Information

Figure S1 **Experiment 1.** Anticipatory activity. Locomotion (A) and rearing behavior (B). Control vs. reward: * *p*<.05, ** *p*<.01. FD vs. FAL: ++ *p*<.01. Day 7 differed from days 8 to 10 in reward rats: # all *p*<.05. Data are expressed as mean+SEM (control = 10, reward = 20).
(TIF)

Figure S2 **Experiment 2.** Anticipatory activity. Locomotion and rearing in the runway maze (A–B). A. Locomotion and rearing in the cage (C–D). Control vs. reward: ** *p*<.01. FD vs. FAL: + *p*<.05, ++ *p*<.01. FD vs. FAL in both groups: xx all *p*<.01. Day 1 differed from days 3 to 7 in both groups: § *p*<.05. Data are expressed as mean+SEM (control = 10, reward = 20).
(TIF)

Figure S3 **Experiment 3.** Anticipatory activity. Locomotion (A) and rearing behavior (B). Control vs. reward: ** *p*<.01. FD vs. FAL in both groups: xx all *p*<.01. Data are expressed as mean+SEM (control = 12, reward = 12).
(TIF)

Figure S4 **Experiment 4.** Anticipatory activity. Locomotion (A) and rearing behavior (B). Control vs. reward: * *p*<.05, ** *p*<.01. FD vs. FAL in both groups: xx all *p*<.01. FD vs. FAL: ++ *p*<.01. Day 7 differed from all FD days in controls, and from day 8 in reward rats: & all *p*<.05. Day 8 differed from day 10 in reward rats: # *p*<.05. Data are expressed as mean+SEM (control = 10, reward = 10).
(TIF)

Figure S5 **Experiment 5.** Anticipatory activity. Locomotion (A) and rearing behavior (B). Control vs. reward: ** *p*<.01. Day 1 differed from days 3 to 7 in reward rats: § *p*<.05. Data are expressed as mean+SEM (control = 10, reward = 10).
(TIF)

Table S1 (DOC)
