# Supplementary figures and images for: Attribution and Expression of Incentive Salience Are Differentially Signaled by Ultrasonic Vocalizations in Rats
Source: PLoS One. 2014 Jul 21;9(7):e102414. doi: 10.1371/journal.pone.0102414 (PMC4105501; doi:10.1371/journal.pone.0102414)

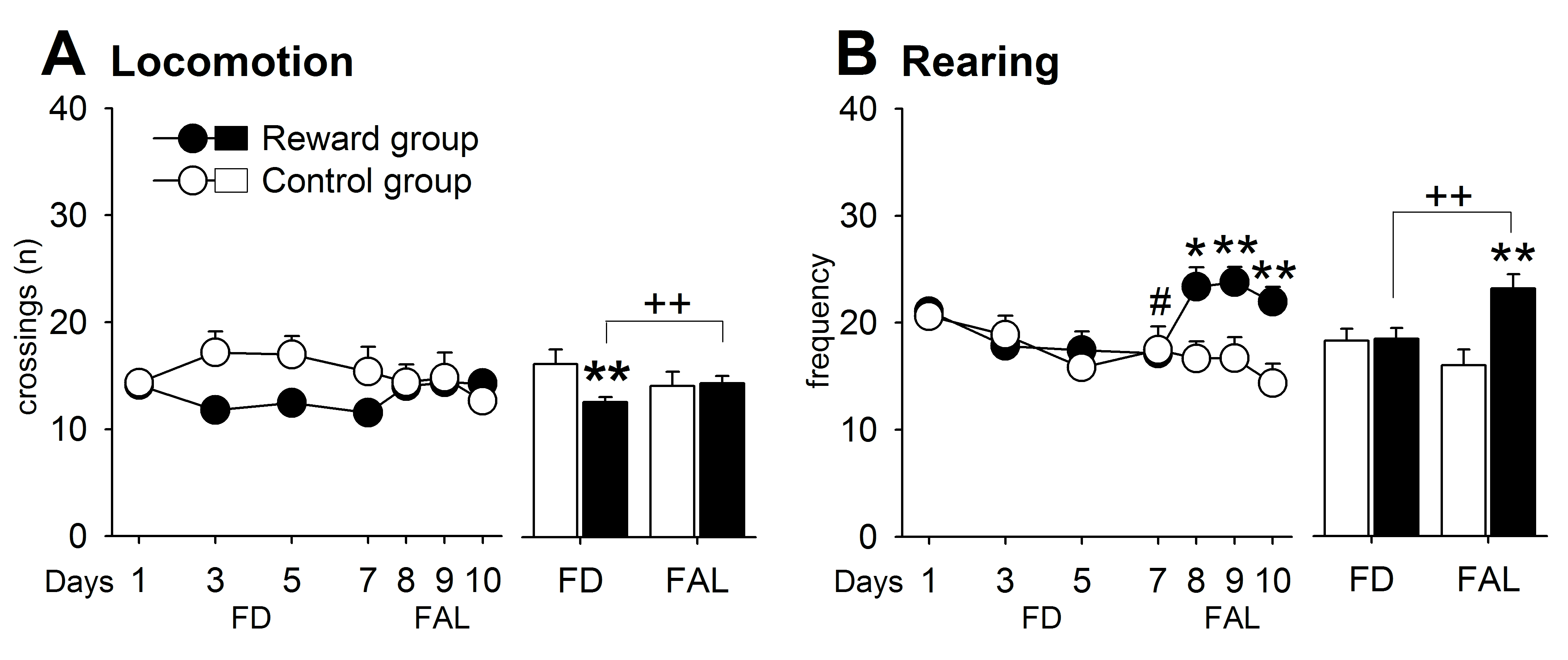

Supplement: Figure S1 — Experiment 1. Anticipatory activity. Locomotion (A) and rearing behavior (B). Control vs. reward: * p<.05, ** p<.01. FD vs. FAL: ++ p<.01. Day 7 differed from days 8 to 10 in reward rats: # all p<.05. Data are expressed as mean+SEM (control = 10, reward = 20). (TIF) [file pone.0102414.s001.tif]

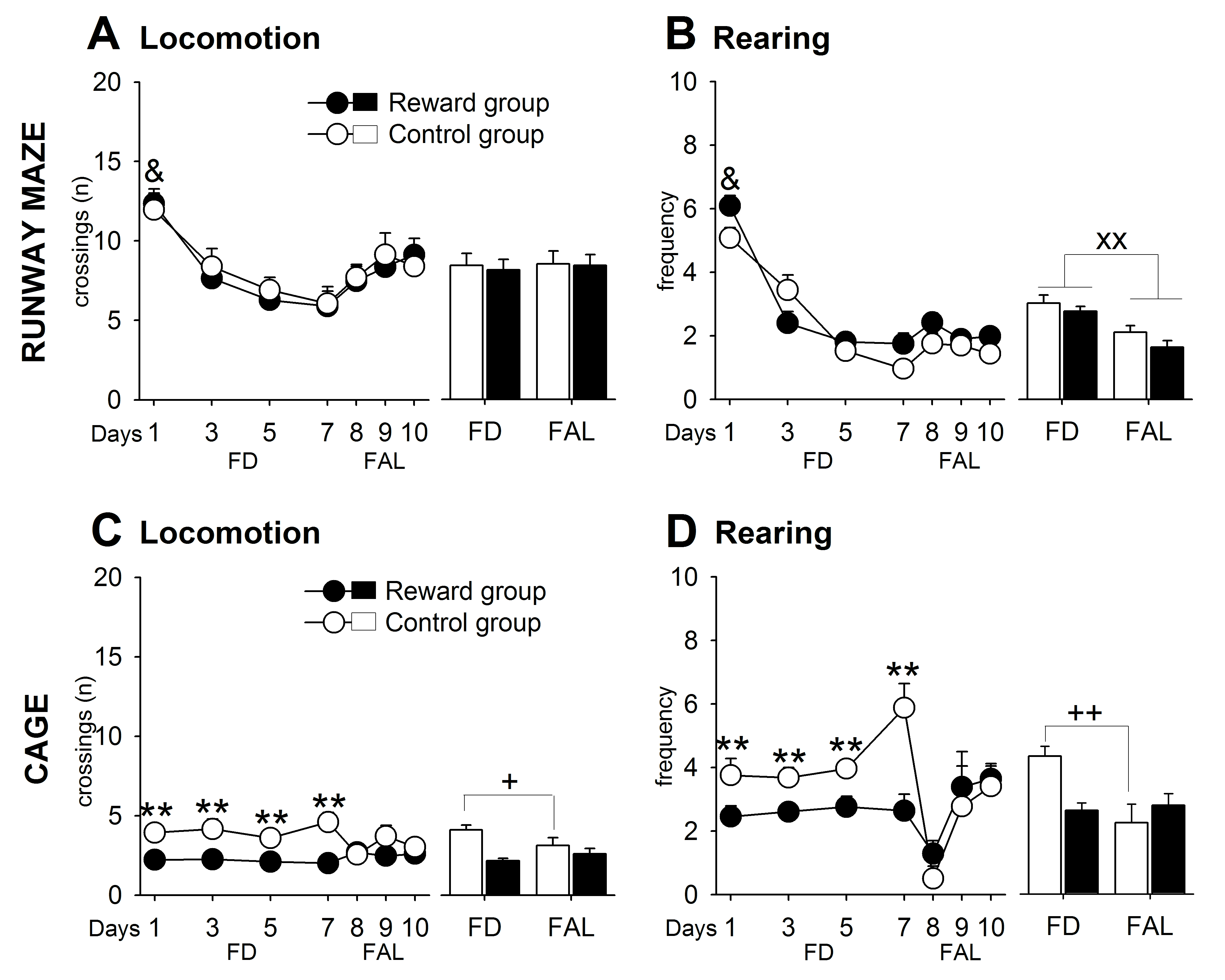

Supplement: Figure S2 — Experiment 2. Anticipatory activity. Locomotion and rearing in the runway maze (A–B). A. Locomotion and rearing in the cage (C–D). Control vs. reward: ** p<.01. FD vs. FAL: + p<.05, ++ p<.01. FD vs. FAL in both groups: xx all p<.01. Day 1 differed from days 3 to 7 in both groups: § p<.05. Data are expressed as mean+SEM (control = 10, reward = 20). (TIF) [file pone.0102414.s002.tif]

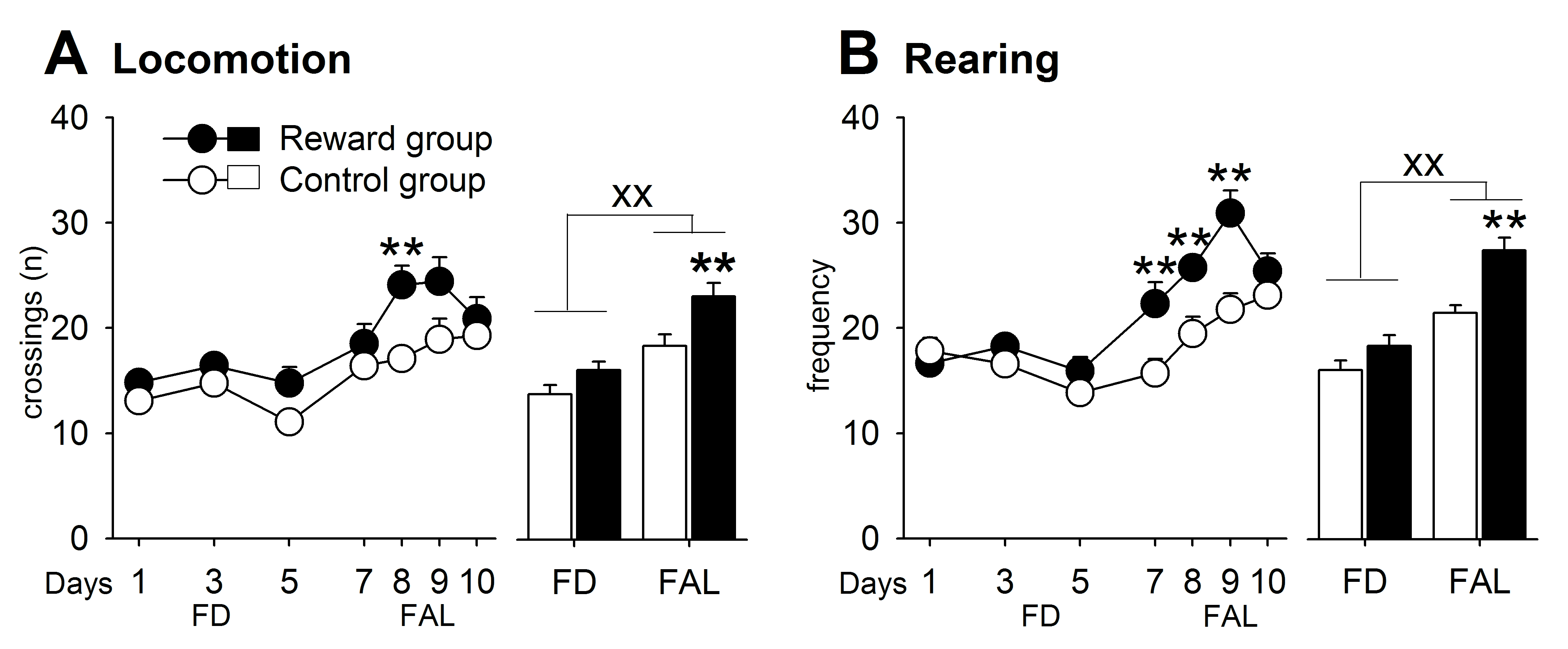

Supplement: Figure S3 — Experiment 3. Anticipatory activity. Locomotion (A) and rearing behavior (B). Control vs. reward: ** p<.01. FD vs. FAL in both groups: xx all p<.01. Data are expressed as mean+SEM (control = 12, reward = 12). (TIF) [file pone.0102414.s003.tif]

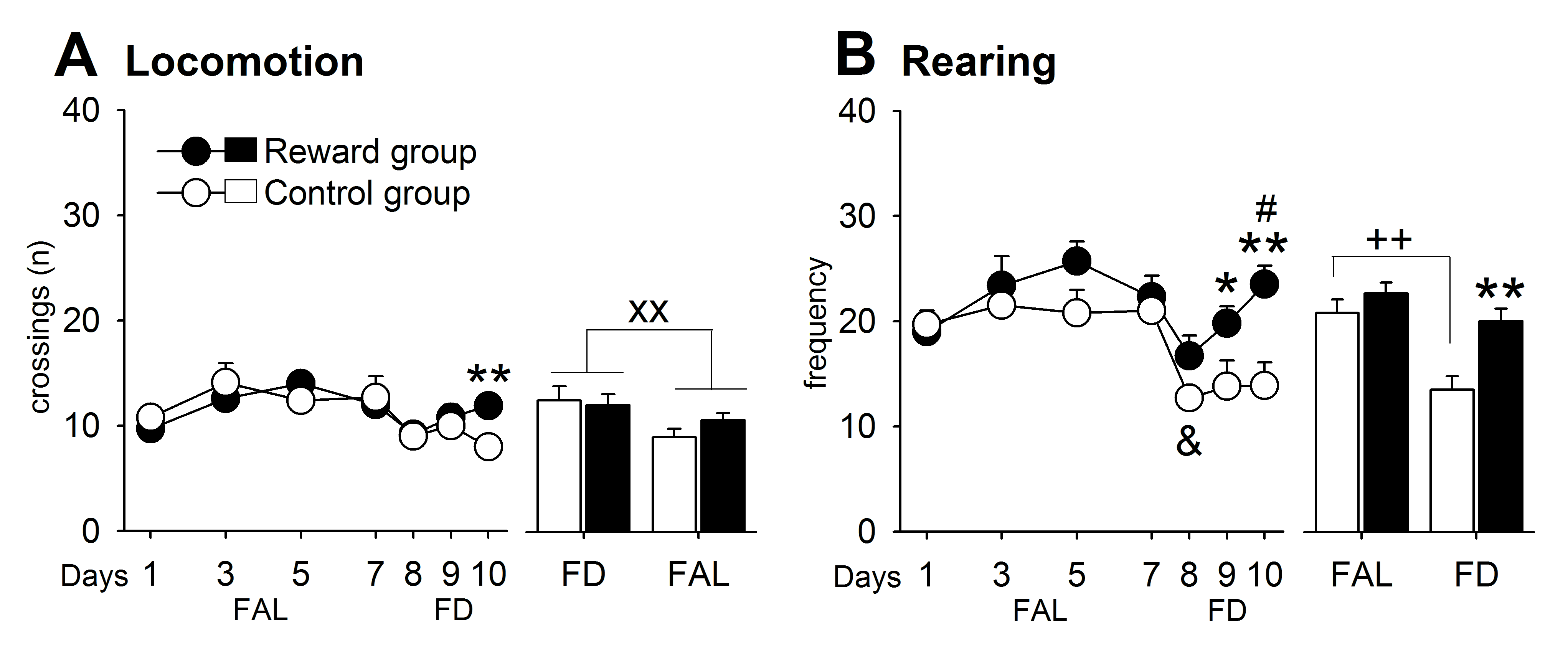

Supplement: Figure S4 — Experiment 4. Anticipatory activity. Locomotion (A) and rearing behavior (B). Control vs. reward: * p<.05, ** p<.01. FD vs. FAL in both groups: xx all p<.01. FD vs. FAL: ++ p<.01. Day 7 differed from all FD days in controls, and from day 8 in reward rats: & all p<.05. Day 8 differed from day 10 in reward rats: # p<.05. Data are expressed as mean+SEM (control = 10, reward = 10). (TIF) [file pone.0102414.s004.tif]

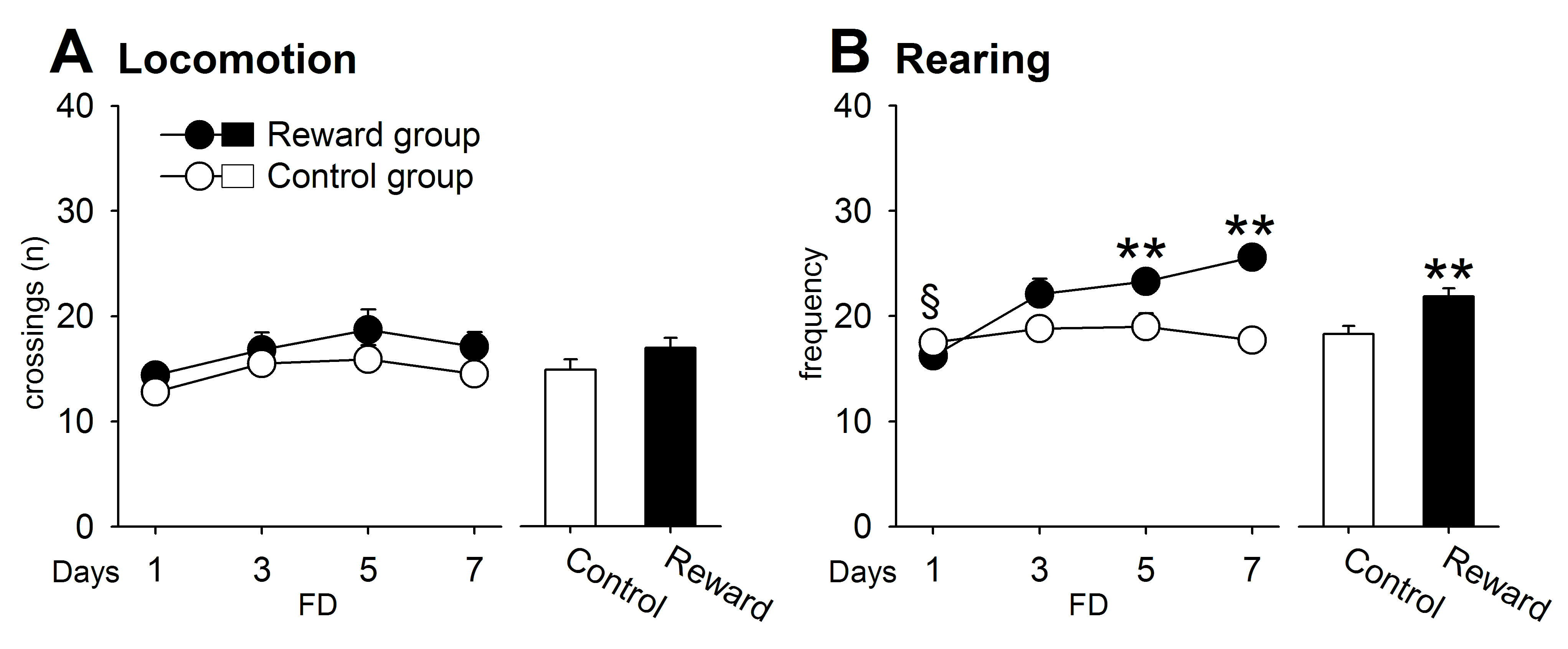

Supplement: Figure S5 — Experiment 5. Anticipatory activity. Locomotion (A) and rearing behavior (B). Control vs. reward: ** p<.01. Day 1 differed from days 3 to 7 in reward rats: § p<.05. Data are expressed as mean+SEM (control = 10, reward = 10). (TIF) [file pone.0102414.s005.tif]
