# Supplementary material for: Attribution and Expression of Incentive Salience Are Differentially Signaled by Ultrasonic Vocalizations in Rats
Source: PLoS One. 2014 Jul 21;9(7):e102414. doi: 10.1371/journal.pone.0102414 (PMC4105501; doi:10.1371/journal.pone.0102414)
Supplement: Table S1 — (DOC) [file pone.0102414.s006.doc]

# Table S1. Time spent (s) digging across experiments

| **Experiment** | **Feeding phases ^1^** | **Control ^2^** | **Reward ^2^** | **Between-groups ANOVA** |
| --- | --- | --- | --- | --- |
| 1 | FD | 4.82±0.58 | 17.32±0.97 | *F*_1,28_=74.845, *p*=.0001 |
|  | FAL | 3.77±0.57 | 2.41±0.34 | *F*_1,28_=4.803, *p*=.037 |
| 2 | FD | 0.30±0.14 | 1.44±0.58 | *F*_1,28_=1.866, *p*=.183 |
|  | FAL | 0.041±0.03 | 0.038±0.04 | *F*_1,28_=0.003, *p*=.957 |
| 3 | FD | 5.25±0.85 | 8.17±1.21 | *F*_1,22_=3.932, *p*=.060 |
|  | FAL | 8.99±1.26 | 8.56±0.70 | *F*_1,22_=0.089, *p*=.769 |
| 4 | FAL | 5.84±0.51 | 4.11±0.95 | *F*_1,18_=2.579, *p*=.126 |
|  | FD | 5.74±1.04 | 2.63±0.86 | *F*_1,18_=5.295, *p*=.034 |
| 5 | FD | 7.63±1.42 | 15.88±2.64 | *F*_1,18_=7.544, *p*=.013 |
| ^1^ FD: food deprived (days 1–7). FAL: food ad libitum (days 8–10). Both phases comprised the same testing days across experiments, except in Experiment 4 in which the testing order was reversed: FAL (days 1–7) and FD (8–10). ^2^ Data represent mean±SEM of cumulative digging (s/min) averaged per feeding phase. | | | | |
